# A study of the controlled degradation of polypropylene containing pro-oxidant agents

**DOI:** 10.1186/2193-1801-2-623

**Published:** 2013-11-20

**Authors:** Celso Luis de Carvalho, Alexandre F Silveira, Derval dos Santos Rosa

**Affiliations:** Universidade Federal do ABC - UFABC, Av do Estado, 5001, Santo André, SP Brazil

**Keywords:** Degradation, Oxidizing, Polypropylene, Polyacetal

## Abstract

Intentional degradation by pro-oxidant agents, many of which are metal-based, can result in uncertainty as to the time of biodegradation. Polyacetal (POM) is a thermoplastic polymer commercially classified as an engineering polymer and contains carbon, hydrogen and oxygen. The depolymerization of POM during processing can enhance thermal decomposition. The aim of this study was to investigate the controlled degradation of polypropylene induced by the degradation of POM or d_2_w®. Mixtures of polypropylene containing different concentrations of POM or d_2_w® were prepared by extrusion. The properties of the mixtures (blends) were evaluated based on the melt index (MFI), tensile properties, Fourier transform infrared spectroscopy (FTIR), Time inductive oxidation (OIT) and Thermogravimetric analysis (TGA). The two additives (POM and d_2_w®) enhanced the oxidative thermal degradation of polypropylene and the degradation of the polypropylene/POM mixture could be controlled by altering the POM concentration.

## Introduction

Packaging waste accounted for 78.81 million tons or 31.6% of municipal solid waste (MSW) in the United States in 2003, 56.3 million tons or 25% of MSW in Europe in 2005, and 3.3 million tons or 10% of MSW in Australia in 2004. In the US, the predominant method of waste disposal is currently landfill packaging, followed by recycling, composting and incineration (Kale et al. 2007). Commodity polymers (polyethylene PE, polypropylene PP, polystyrene PS, polyvinyl chloride PVC and polyethylene terephtalate PET) prevail in packaging applications (PlasticsEurope 2011) and polyolefins are increasingly being used in new applications (Gahleitner 2011). An excellent way of producing degradable polyethylene is to mix this polymer with pro-oxidant additives that can effectively improve the degradability of these materials (Roy et al. 2007). Intentional degradation by pro-oxidant agents, many of which are metal-based (Roy et al. 2007), has generated uncertainties in the evaluation of biodegradation (European Bioplastics 2012) and several surveys it is claimed that polyolefins (PE, PP) is an inert polymer with good resistance to microorganisms (Albertsson 1978,2003). The controlled degradation of polypropylene has been used in rheological control by distributing and reducing the molar mass of organic peroxides in reactive extrusion (Rocha et al. 1994; Kim 1996). Polyacetal (POM) is a thermoplastic polymer that is susceptible to thermal decomposition (depolymerization) (Cottin et al. 2000). The objective of this study was to investigate the controlled degradation of polypropylene induced by the degradation of an organic oxidizing agent (POM) in extrusion. The additive d_2_w®, a commercial metal-based pro-oxidant, was used for comparison.

## Materials and methods

### Materials

Isotactic polypropylene (iPP) H603 (density: 0.905 g/cm^3^; MFI: 1.5 g/10 min) was used in granulated form, as supplied by Braskem (Triunfo, RS, Brazil). The polyacetal copolymer (density: 1.42 g/cm^3^; MFI: 14.0 g/10 min) was used in powder form as supplied by Ticona (São Paulo, SP, Brazil). The commercial pro-oxidant additive d_2_w® was supplied by RES Brazil (São Paulo, SP, Brazil).

### Methods

#### Preparation of the mixtures

The additive d_2_w® was incorporated in granular form and POM in powder form. The incorporation of POM or d_2_w® into polypropylene (PP) initially involved homogenization in Drais with a load capacity of ~100 g of material. The mixing time was ~30 s. Blends of PP with POM or d_2_w® were prepared as shown in Table 1 using a single screw extruder fitted with a 25 mm diameter screw, a heating cylinder with an L/D ratio of 25:1 and four wire screens in series (60, 150, 150 and 100 mesh) to maximize the homogeneity. The extrusion conditions were 220°C, 250°C and 250°C for the first, second and third zones, respectively.

**Table 1:** **The polymer/additive mixtures used in this study**

Code	PP	POM	d_2_w^®^	Code	PP	POM	d_2_w^®^
PP_1_	100*	0*	0*	PP_5_	100	0	2
PP_2_	100	1	0	PP_6_	100	3	0
PP_3_	100	0	1	PP_7_	100	0	3
PP_4_	100	2	0	PP_8_	100	10	0

(*) Amount expressed in parts per hundred of resin (phr).

### Analysis

#### Melt flow index (MFI)

The MFI was determined in a plastometer (model 7023.000, CEAST, Ohio, USA) according to ASTM D-1238 (ASTM 2004). The test conditions were set at a load of 2,160 kg and a temperature of 230°C for all mixtures.

#### Mechanical tests

Type IV specimens (ASTM D-638-10) (ASTM 2010) were injected into a model PIC-BOY 22 machine (Petersen & Cia Ltda, São Paulo, SP, Brazil) with an injection capacity of 22 g of polystyrene. The total cycle time was 30 s and the temperatures of zones 1 (injection nozzle), 2 and 3 were 220°C, 220°C and 180°C, respectively. The tensile test was done in a universal testing machine (model 5569, Instron), according to ASTM D638-10, at a test speed of 25 mm/min and cell load of 50 kN. The tensile strength at break and elastic modulus were determined.

#### Fourier transform infrared spectroscopy (FTIR)

Films 30 ± 2 μm thick were prepared at 190°C with a compression pressure of 2000 psi and compression time of 80 s. FTIR measurements were obtained using a Varian 660-IR FT-IR spectrometer operated in transmittance mode. Thirty-two scans were obtained in triplicate from 4000 cm^-1^ to 400 cm^-1^ at a resolution of 4 cm^-1^. The influence of POM and d_2_w® on polypropylene oxidation was determined from the spectra by calculating the carbonyl (CI) and hydroxyl (HI) indices based on the relationships CI = A_1725_/A_2722_ and HI = A_3500_/A_2722_, respectively.

#### Differential scanning calorimetry (DSC)

DSC was done in a TA Instruments calorimeter at a nitrogen flow of 50 ml/min. Approximately 10 mg of each sample was heated, cooled and heated again over a temperature range of 25–250°C at a heating and cooling rate of 10°C/min. The melting temperature (Tm), crystallization temperature (Tc) and degree of crystallinity were calculated using the enthalpy of fusion values of 209 J.g^-1^ and 306 J.g^-1^ for 100% crystalline polypropylene and polyacetal, respectively (Canevarolo 2003; Kumar et al. 1995).

#### Oxidation induction time (OIT)

The OIT was determined by exposing ~10 mg of each mixture to a nitrogen flow of 50 ml/min and a heating rate of 20°C/min. An oxygen flow of 50 ml/min was used after melting at 200°C.

#### Thermogravimetric analysis (TGA)

TGA was done in equipment from TA Instruments. Approximately 10 mg of each mixture was placed in a nitrogen atmosphere and heated at a rate of 10°C/min over a temperature range of 25–550°C. The nitrogen flow over the measurement cell was 50 ml/min. The activation energy of degradation (Ea) was determined according to ASTM E1641 (ASTM 2007).

## Results and discussion

### Melt flow index (MFI)

**Figure 1:**
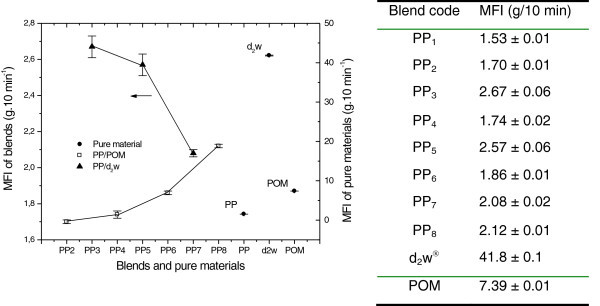
**MFI for pure materials (PP, POM, d**_**2**_ **w®) and blends (PP/POM, PP/d**_**2**_ **w®).** The points are the mean ± SD of 6 determinations. **b)** Average results and their respective estimated standard deviation of the melt index of the compositions and pure materials.

The MFI is inversely related to sample viscosity and can be used to estimate the interaction between the phases in polymer mixtures (Huang et al. 2003). Figure 1 shows that pure PP had the lowest MFI of all samples, indicating that it had the highest viscosity under the test conditions. The MFI increased as the content of POM in the mixtures increased (PP_2_, PP_4_, PP_6_ and PP_8_) and probably reflected the immiscibility between the phases (PP/POM) since the variation in the measurements was proportional to the mass of POM in the PP/POM mixture (blend) (Huang et al. 2003). In polypropylene mixtures PP_3_, PP_5_ and PP_7_ the flow behavior was reversed, i.e., the MFI decreased as the concentration of d_2_w® increased. Partial miscibility between the vehicle solution for the additive d_2_w® and the polypropylene matrix could explain this result, although the possibility of degradation of the matrix and dispersed phase to generate cross-linked additive carrier material (dispersed phase) and polypropylene (matrix) should also be considered (Huang et al. 2003; Waldman and De Paoli 1998; Bouhelal et al. 2010).

### Tensile testing

**Figure 2:**
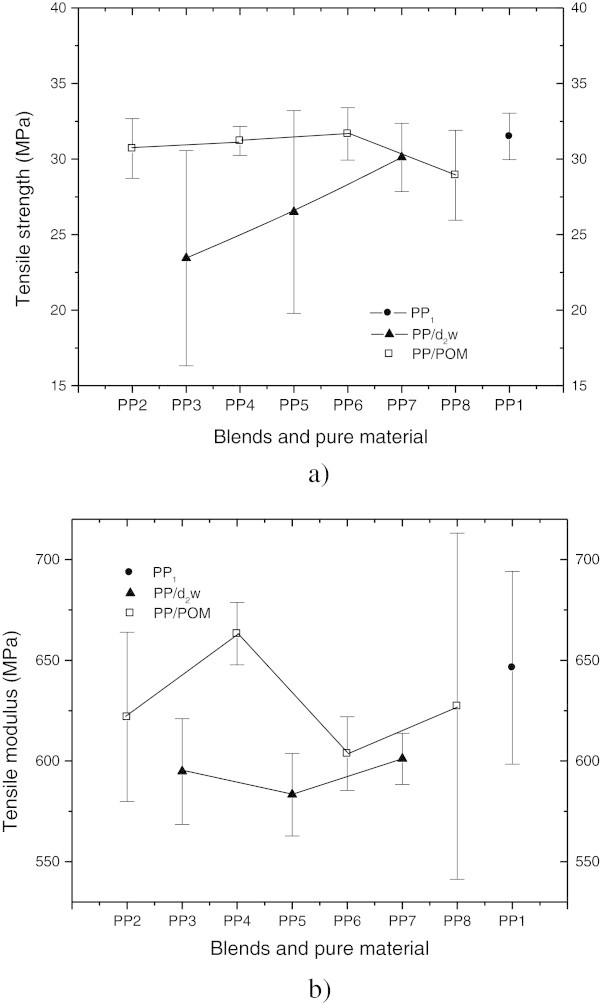
**a) Tensile strength and b) elasticity modulus of pure polypropylene (PP**_**1**_ **) and blends with POM and d**_**2**_ **w®.** The points are the mean ± SD of 6 determinations.

Figure 2 shows the tensile modulus of elasticity and flow of polypropylene and blends with d_2_w® or POM. Mixtures containing d_2_w® showed larger standard deviations than those containing POM. This difference probably reflected the degree of dispersion of the additive in the polypropylene matrix since d_2_w® was incorporated into polypropylene in granular form while POM was incorporated in powder form. This conclusion suggests interaction between the degradative processes associated with polypropylene and POM during sample preparation. The differences in the tensile strength of the two compositions (Figure 2a) most likely reflect variations in the inherent mechanical strength of the carrier material and the additive d_2_w®. Likewise, differences in the modulus of elasticity (Figure 2b) reflect variations in the stiffness of the additive d_2_w® and POM (Huang et al. 2003). The standard deviations for the modulus of elasticity of pure polypropylene and the PP_8_ mixture were also high. The presence of tiny, randomly distributed bubbles in these two preparations could have contributed to this high standard deviation; differences in the composition of the samples are unlikely to be a cause of this variation.

### Infrared spectroscopy (FTIR)

**Figure 3:**
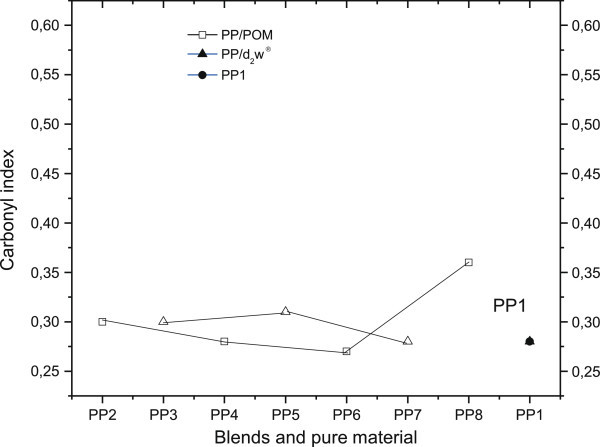
**Carbonyl indices of pure material (PP**_**1**_ **) and PP/POM and PP/d**_**2**_ **w® blends.**

**Figure 4:**
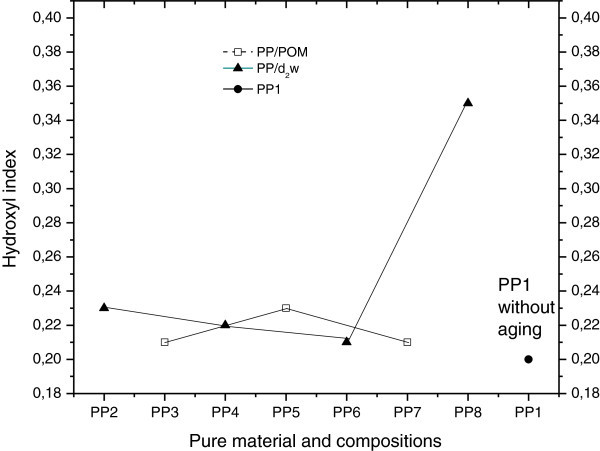
**Hydroxyl indices of pure material (PP**_**1**_ **) and PP/POM and PP/d**_**2**_ **w® blends before and after thermal aging.**

Absorbance in the region of 1725 cm^-1^ and 3500 cm^-1^ indicated the presence of carbonyl and hydroxyl groups (De Paoli 2008; Cáceres and Canevarolo 2009), respectively, and the absorbance peak at 2722 cm^-1^ was related to angular molecular vibrations in CH and axial molecular vibrations in CH_3_, as suggested elsewhere (Cáceres and Canevarolo 2009; Babetto and Canevarolo 2002; Rabello and White 1997; Garton et al. 1978). Comparison of these absorbances can be used to normalize IR spectra since these peaks are insensitive to the oxidative degradation of pure polypropylene (Wang et al. 2011). Figure 3 and Figure 4 show variation in the carbonyl (CI) and hydroxyl (HI) indices, respectively. The CI index showed increased formation of carbonyls in mixture PP_8_, whereas PP_3_ and other mixtures containing d_2_w® (PP_5_ and PP_7_) were quite stable in this parameter. The HI index showed similar behavior to the CI index shown in Figure 3, but mixture PP_8_ had high hydroxyl formation, probably indicating greater susceptibility to the formation of nOH (acetic acid) (Duan et al. 2006) during processing, with absent or low oxygen supply during extrusion of the mixtures and injection of the samples. Together, these results indicate that the addition of d_2_w® did not enhance the degradation of polypropylene, whereas the presence of POM resulted in greater thermal degradation of polypropylene that was proportional to the content of POM and greater than the concentration of POM in mixture PP_6_ (3% by weight).

### Differential scanning calorimetry (DSC)

**Figure 5:**
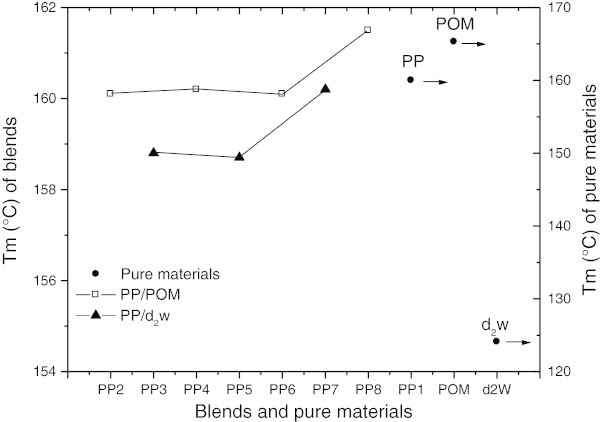
**Crystalline melting temperature (Tm) of pure materials (PP, POM and d**_**2**_ **w®) and their blends (PP/POM and PP/d**_**2**_ **w®).**

**Figure 6:**
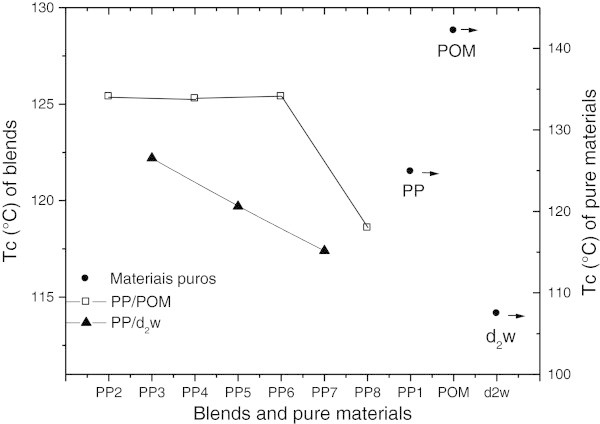
**Crystallization temperature (Tc) of the pure materials (PP, POM and d**_**2**_ **w®) and their blends (PP/POM and PP/d**_**2**_ **w®).**

Table 2 shows the thermal properties of the pure materials (PP_1_, POM and d_2_w®) and mixtures (blends) (PP/d_2_w® and PP/POM). Figure 5 and Figure 6 show the crystalline melting temperatures (Tm) and crystallization temperatures (Tc), respectively. There was a trend towards a higher Tm for all mixtures containing POM and d_2_w®. In mixtures containing d_2_w® (PP_3_, PP_5_ and PP_7_) there was a reduction in Tc that was proportional to the d_2_w® concentration, such that the Tc was lower than for pure polypropylene. In PP/POM blends (PP_2_, PP_4_, PP_6_ and PP_8_) the Tc of PP_2_, PP_4_ and PP_6_ stabilized at ~5°C above the Tc of pure polypropylene. This is a desirable result in processing by injection because it allows a reduction in the cooling time. The Tc of the PP_8_ blend was lower than for the other mixtures and also in relation to pure POM. This finding suggests that morphological changes in POM are probably the result of its thermal degradation.

**Table 2:** **Thermal properties of pure materials (PP**_**1**_ **, POM and d**_**2**_ **w®) and blends of PP/POM and PP/d**_**2**_ **w®**

Compositions	Tm (°C)	ΔHm (J/g)	Tc (°C)	ΔHc (J/g)	Crystallinity (%)
PP_1_ 100%	160.0	89.7	124.9	96.7	42.9
PP_2_ 100/1	160.1	93.6	125.4	90.7	44.8
PP_3_ 100/1	158.8	85.8	122.2	94.3	41.1
PP_4_ 100/2	160.2	96.9	125.3	93.6	46.4
PP_5_ 100/2	158.7	87.2	119.7	94.8	41.7
PP_6_ 100/3	160.1	97.8	125.4	93.9	46.8
PP_7_ 100/3	160.2	80.8	117.4	86.7	38.7
PP_8_ 100/10	161.5	89.3	118.6	95.6	42.7
POM 100%	165.3	138.5	142.2	136.5	42.5
d_2_w® 100%	124.1	114.3	107.5	116.5	-

**Figure 7:**
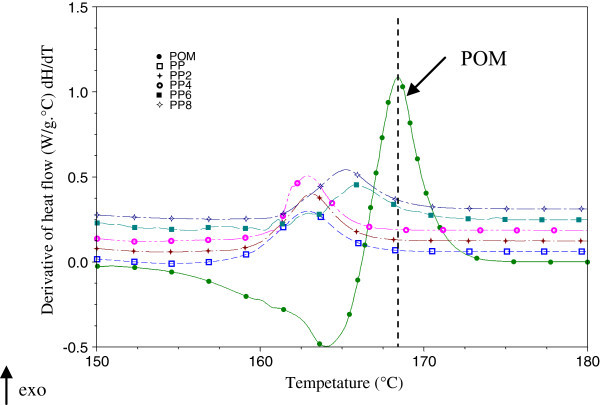
**Derivative of heat flow in the crystallization of pure materials (PP**_**1**_ **and POM) and PP/POM blends (PP**_**2**_ **, PP**_**4**_ **, PP**_**6**_ **and PP**_**8**_ **) without thermal aging.**

**Figure 8:**
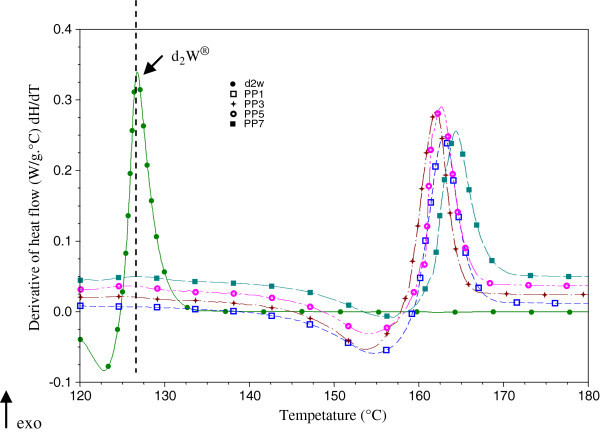
**Derivative of heat flow in the crystallization of pure materials (PP**_**1**_ **and d**_**2**_ **w®) and blends of PP/d**_**2**_ **w® (PP**_**3**_ **, PP**_**5**_ **and PP**_**7**_ **) without thermal aging.**

Figure 7 and Figure 8 show the heat flux derived from the crystallization of PP/POM (PP_2_, PP_4_, PP_6_ and PP_8_) and PP/d_2_w® (PP_3_, PP_5_ and PP_7_). Mixtures containing POM showed additional peaks between the peaks of pure POM and polypropylene. Degradation reactions usually appear as endothermic processes and exothermic behavior is generally a response to depolymerization (Canevarolo 2003; De Paoli 2008). Indeed, an endothermic response was observed in the curve of the derivative of heat flow during the crystallization of pure POM (Figure 7). The coexistence of these two processes in PP/POM mixtures suggests a change in the Tc and the formation of volatiles that are not detected by thermogravimetric analysis at temperatures below 200°C. During the crystallization of mixtures of PP/d_2_w® there is no heat flow between the peaks of the pure materials (PP and d_2_w®).

### Oxidation induction time (OIT)

**Figure 9:**
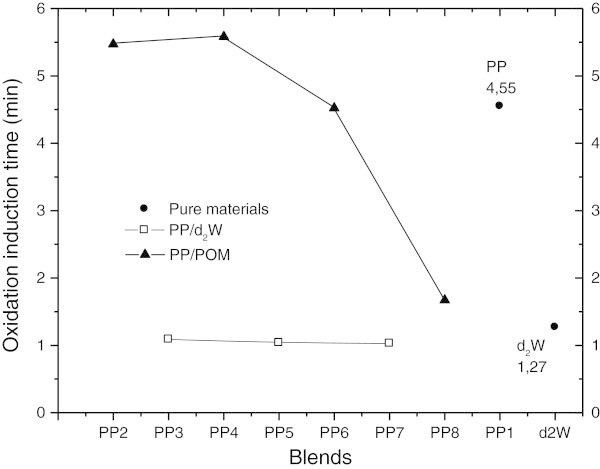
**Oxidation induction time for pure materials and blends.**

The oxidation induction time (OIT) is an accelerated aging test that allows comparison of the relative resistance of materials to thermal oxidation. Table 3 and Figure 9 provide the OIT values for the pure materials and blends studied. The additive d_2_w® accelerated the oxidation of polypropylene in the presence of oxygen. However, there was little change in the OIT values of blends containing different amounts of d_2_w® (PP_3_, PP_5_ and PP_7_), i.e., the OIT values essentially reflected the amount of pure additive (d_2_w®) present in the mixtures. This finding indicates that there is little margin for controlling the thermo-oxidation of polypropylene during processing since all samples were processed under the same condition. In addition, changes in morphology arising from processing interfere with the diffusion of volatile degradation products.

**Table 3:** **Oxidation induction time for pure materials and blends**

	Pure materials and blends									
	PP_1_	PP_2_	PP_3_	PP_4_	PP_5_	PP_6_	PP_7_	PP_8_	d_2_w®	POM
Time (min)	4.55	5.47	1.09	5.58	1.04	4.52	1.03	1.67	1.27	< 1

In PP/POM blends, there was a marked decrease in the OIT values from blend PP_6_ onwards. In contrast, there was an increase in the OIT values of blends PP_2_ and PP_4_, i.e., a stabilizing (antioxidant) effect. A similar delay in the kinetics of degradation was observed in the absence of oxygen in the TGA of these two blends, i.e., the Ti of the blends was greater than that of pure polypropylene (PP_1_). The OIT and TGA results indicated that d_2_w® concentrations ≥2% increased the thermal stability of the blends. In the case of POM, there was a decrease in the stabilizing synergistic effect at concentrations up to 3%; at higher concentrations, POM had an oxidizing effect on polypropylene.

The oxidation of a polymer involves a complex chain of reactions that involves many steps such that the overall Ea is the sum of the energies of activation of individual stages. In this chain of reactions there may be temperature ranges in which deviations from Arrhenius’ law can be neglected, e.g., with blends PP_2_ and PP_4_. The oxidation of polypropylene (in powder form) has been referred to as non-homogeneous (heterogeneous) kinetics that is characterized by chemiluminescence (Celina and George 1995). This oxidation is based on a model containing small numbers of localized zones (amorphous regions) in which oxidation occurs at a high rate and from where it spreads to other regions. The presence of stabilizers retards the diffusion of volatile degradation products for a short period of time known as the induction period. Even using sensitive techniques involving photon emission, such as chemiluminescence, the investigation of this phenomenon over such a short timescale is a difficult task, even though the Ea is higher in this period (Celina and George 1993). Several studies (Wang et al. 2011; Bouhelal et al. 2010; Groening and Hakkarainen 2002; Albertsson and Hakkarainen 2008) have shown that the decomposition of hydroperoxides in polypropylene leads to the formation of volatile products and that water is a major product of degradation but does not interfere with the spread of oxidation. During this period, generally only a decrease in polymer molecular mass is observed, along with the formation of volatile, low molecular mass products. Eriksson (Eriksson et al. 2001) suggested that following the formation of peracids by the oxidation of formaldehyde, the spreading of oxidation is favored by the gas phase and that the relatively low reactivity of formaldehyde allows greater diffusion to more distant regions.

### Thermogravimetric analysis (TGA)

**Figure 10:**
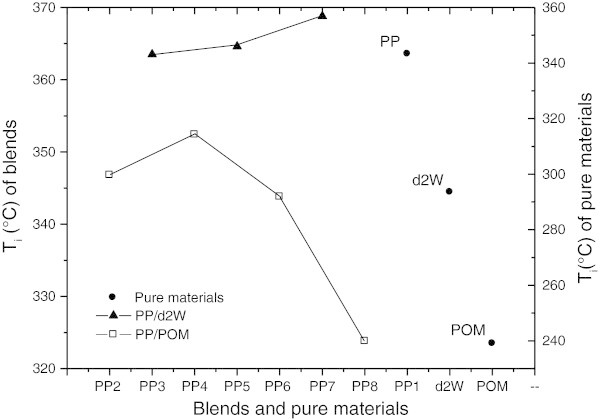
**Onset degradation temperature (Ti) determined at a rate of 10°C/min for pure materials and blends.**

Figure 10 shows the temperature for the onset of degradation (Ti) of the pure materials and blends. The variation in the Ti of blends compared to pure polypropylene may reflect the formation of volatile components and their diffusion in the polymeric matrix. The pure materials (POM and d_2_w®) had a lower Ti than polypropylene, with POM showing the lowest value. The Ti of the blends were not lower than those of POM and d_2_w®, except for blend PP_8_. The values of Ti varied with the concentration of d_2_w® and POM in polypropylene. In the case of d_2_w®, increasing the concentration of the additive led to an increase in Ti and therefore had a stabilizing effect. This enhanced response can be attributed to the greater thermal stability of the vehicle for d_2_w® relative to polypropylene. The MFI corroborated this stabilizing effect. Increasing the concentration of POM led to an increase in Ti up to the value seen with blend PP_4_, after which the Ti stabilized up to PP_6_ and then decreased in blend PP_8_.

Together, these results indicate that the addition of d_2_w® to polypropylene increased the thermal stability in a manner dependent on the concentration of additive. The low production of volatile components and the reduced mobility (diffusion capacity) of the polypropylene matrix were probably important factors in this thermal stability. The addition of POM to polypropylene resulted in synergistic and antagonistic effects (stabilization and degradation), with the extent of stabilization and degradation depending on the amount of volatile components produced.

**Figure 11:**
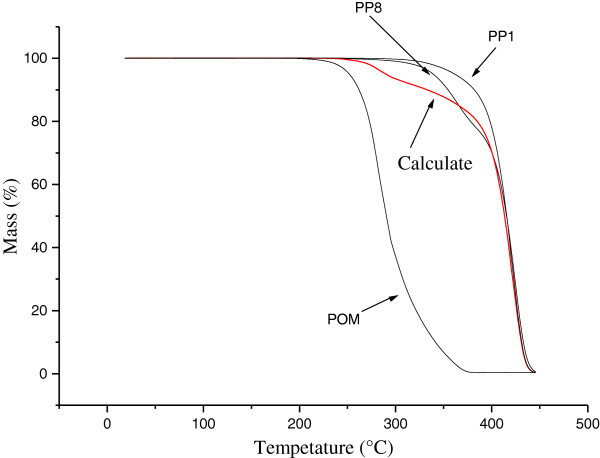
**Experimental curves for changes in mass versus time for blend PP8 and pure materials (PP and POM).** The theoretical (calculated) for blend PP8 is also shown.

Figure 11 shows the change in mass as a function of temperature for blend PP_8_ and for the pure materials (PP_1_ and POM); a theoretical curve calculated curve calculated based on the weighted average of the experimental curves of the pure materials (PP and POM) is also shown. The region of the curve for blend PP_8_ close to the experimental curve of pure polypropylene (PP_1_) may reflect a greater stabilizing effect of polypropylene. The stabilizing effect decreases as the loss of mass increases and the theoretical curve approximates the observed curve, probably because of competition with degradation reactions. The region beyond the point where the theoretical and observed curves cross corresponds to additional degradation by shifting the equilibrium of the degradation reactions of the matrix phase (PP) induced by the dispersed phase (POM).

**Figure 12:**
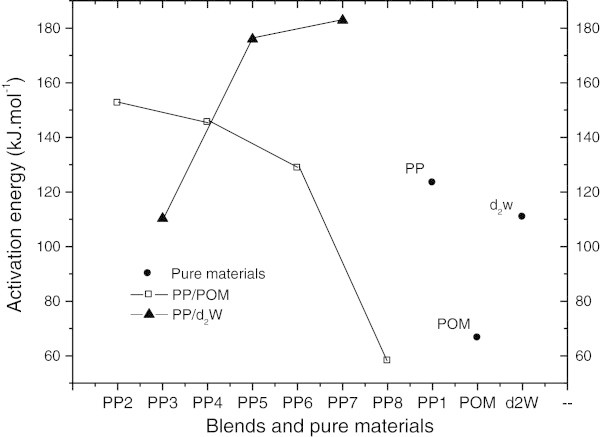
**Energy of activation (Ea) for pure materials (PP, POM and d**_**2**_ **w®) and blends of PP/POM and PP/d**_**2**_ **w®.**

Table 4 and Figure 12 show the energy of activation (Ea) of the pure materials and blends. Mixtures containing d_2_w® and POM showed opposite changes in Ea as a function of the additive concentration in the polypropylene matrix. Ea may be regarded as the energy needed to cause the diffusion of one mole of atoms such that high Ea results in a relatively small diffusion coefficient (Callister 2006). In the blends studied here, the low mobility of pro-oxidant derivatives of d_2_w® attached to a polymeric carrier material may have increased the concentration of additive thereby enhancing the Ea. For blends of PP/POM, the formation of volatile, low molecular mass products such as formaldehyde derived from POM could explain the decrease in Ea with increasing POM concentration. The propagation of oxidation at high temperatures in stabilized polypropylene involves the gas phase (Eriksson et al. 2002; Eriksson et al. 2001; Celina et al. 2006). Among the products generated by the degradation of PP, e.g., water, ethylene, isobutylene and acetic acid, formaldehyde is the one that most likely contributes to the propagation of oxidation via the gas phase in stabilized polypropylene (Eriksson et al. 2002). This conclusion agrees with the calculated curve for blend PP_8_ shown in Figure 10 that suggests a synergistic effect of degradation. The low Ea value for blend PP_8_ (lower than pure POM) is also suggestive of synergism.

**Table 4:** **Activation energy (Ea) and correlation coefficients for pure materials and blends**

Blends	PP_2_	PP_3_	PP_4_	PP_5_	PP_6_	PP_7_	PP_8_
Ea (kJ.mol^-1^)	152.6	110.3	145.5	175.9	128.8	182.8	58.2
r	−0.997	**-** 0.950	- 0.995	**−**0.996	−0.944	**−**0.985	−0.874
Pure materials	PP		POM		d_2_w®		
Ea (kJ.mol^-1^)	123.4		66.6		110.9		
r	−0.937		−0.880		−0.995

## Conclusion

The addition of POM or d_2_w® promoted the oxidative thermal degradation of polypropylene (PP), with the extent of degradation being regulated by the POM concentration in PP/POM blends. At concentrations <3% (w/w), POM enhanced the thermal stabilization of polypropylene under the conditions investigated, whereas at concentrations >3% POM stimulated the oxidation of polypropylene. These results suggest that the POM with a concentration >3% (w/w), may act as a pro-oxidant agent of the PP, and the synergistic effect of degradation can be maximized by increasing the miscibility at the interface of the blend PP/POM.
